# A novel hepatocellular carcinoma-specific mTORC1-related signature for anticipating prognosis and immunotherapy

**DOI:** 10.18632/aging.204862

**Published:** 2023-08-16

**Authors:** Erbao Chen, Yuqian Mo, Jing Yi, Jie Liu, Ting Luo, Zheng Li, Zewei Lin, Yibing Hu, Zhilin Zou, Jikui Liu

**Affiliations:** 1 Department of Hepatobiliary and Pancreatic Surgery, Peking University Shenzhen Hospital, Shenzhen, Guangdong, China; 2 School of Public Health, Guangdong Medical University, Zhanjiang, Guangdong, China; 3 Operating Room, Peking University Shenzhen Hospital, Shenzhen, Guangdong, China; 4 Department of Ophthalmology, Affiliated Eye Hospital of Wenzhou Medical University, Wenzhou, Zhejiang, China; 5 Breast and Thyroid Surgery, Peking University Shenzhen Hospital, Shenzhen, Guangdong, China

**Keywords:** mTORC1, tumor microenvironment, hepatocellular carcinoma, immunotherapy, prognosis

## Abstract

Tumor oncogenesis, cancer metastasis, and immune evasion were substantially impacted by the mammalian target of the rapamycin complex 1 (mTORC1) pathway. However, in hepatocellular carcinoma (HCC), no mTORC1 signaling-based gene signature has ever been published. mTORC1 scores were computed employing a single sample gene set enrichment analysis based on databases including the International Cancer Genome Consortium (ICGC) and The Cancer Genome Atlas (TCGA). The PAG1, LHFPL2, and FABP5 expression levels were obtained to construct a mTORC1 pathway-related model. In two databases, the overall survival (OS) rate was shorter for high-mTORC1 score patients compared to those with low scores. The activation of TFs in the group with high risk was enhanced, such as the HIF-1 pathway. Additionally, it was discovered that a high mTORC1 score was linked to an immune exclusion phenotype and enhanced immunosuppressive cell infiltration. Notably, it was discovered that high-mTORC1 scores patients had poorer immunotherapeutic results and might not gain benefit from immunotherapy. When compared to the low HCC metastatic cell lines, the high HCC metastatic cell lines have overexpressed levels of PAG1, LHFPL2, and FABP5 expression. The expression of PAG1, LHFPL2, and FABP5 was inhibited by the MAPK and mTORC1 pathway inhibitors. Our study identified mTORC1 score signature can aid in the development of individualized immunotherapy protocols and predict the HCC patients’ prognoses.

## INTRODUCTION

Globally, the third leading cause of fatalities related to cancer is hepatocellular carcinoma (HCC), a type of aggressive cancer [1]. Although many treatments, such as immunotherapy and targeted therapy, achieved significant clinical breakthroughs in clinical practice, some patients still fail to benefit [2]. In recent years, the relationship between classic cell signaling and immunity has become an increasingly interesting topic. Therefore, identifying a model with high predictive ability is required to guide treatment and enhance the HCC patients’ prognoses, particularly, to predict the response of immunotherapy.

Traditional experimental approaches for cancer research are focusing on one single gene biomarker during cancer development or progression. Recently, it would be expected that a gene set-based signature model would be more predictive. Several multi-gene signatures have been developed by other researchers to predict clinical results and to aid in clinical decision-making for HCC patients. For example, a tumor-based gene signature [3], A Starvation-Based 9-mRNA Signature [4], and lipid metabolism-related lncRNAs [5] have been investigated to construct prognostic gene signatures in HCC. However, a systematic exploration of crucial signaling pathway-related genes has the potential to identify HCC subtypes with promising implications for predicting prognosis and responding to treatment.

Atypical serine/threonine protein kinase named as mammalian target of rapamycin (mTOR) forms mTORC1 and mTORC2 complexes through binding to different proteins [6]. Growth factors and nutrients (amino acids, fatty acids, lipids, and glucose) are the primary determinants of mTORC1 activation. The phosphatidylinositol 3-kinase (PI3K) /AKT signals are activated by nutrients via mTORC1 translocation to the lysosomal surface from the cytoplasm [7, 8]. The mTOR pathway is highly conserved over the course of evolution and regulates multiple biological processes including cell proliferation, metabolism, survival, and immune regulation [9]. Multiple earlier studies have demonstrated that mTORC1 is remarkably linked to various malignant cancers, including gastric, liver, and breast cancers [10, 11]. The other studies have explored several regulatory pathways underlying the mTOR pathway mediating angiogenesis and tumor immunity [12, 13]. Hyperactivated mTORC1 enhances hypoxia-inducible factor-1α at several stages, which stimulates angiogenesis by improving the transcription of pro-angiogenic factors like vascular endothelial growth factor (VEGF) and transforming growth factor-α in feedback. According to Yang et al., inhibiting the mTORC1/STAT3 signaling pathway decreases tumor angiogenesis [14, 15]. mTORC1 activity promotes c-MYC levels to enhance the expression of glycolytic enzymes and transporters during initial T-cell activation [16]. Loss of mTORC1 significantly impairs the maturation of NK cells in the early stages [17]. Terminal maturation of NK cells also necessitates Tsc1-dependent negative regulation of mTORC1 activation [18]. Villanueva et al. provided the first in-depth description in 2008 of how the mTOR pathway is aberrantly activated as HCC progresses [19]. Around 50% of cases of human HCC, according to the authors, had the mTORC1 cascade activated. Importantly, The Cancer Genome Atlas (TCGA) search gained an in-depth understanding of the dysregulation of the mTORC1 pathway throughout hepatocarcinogenesis. Thus, a mTORC1 signaling-based signature might be developed and employed to anticipate the prognosis and HCC patients’ responsiveness to immunotherapy.

In this study, we comprehensively investigated all mTORC1 signaling-related genes and developed a prognostic risk model for the mTORC1 pathway based on databases of the International Cancer Genome Consortium (ICGC) and TCGA. Then we characterized the molecular pathways, transcriptional factors, and infiltrating immune cells for mTORC1 signaling-related signature, and assessed its predictive ability for immunotherapy. To discover the mTORC1 signaling-immunity mechanism, the current research offers a fresh viewpoint.

## MATERIALS AND METHODS

### Patients and datasets

TCGA-LIHC (http://portal.gdc.cancer.gov/repository) provided the transcriptome information and corresponding HCC patients’ clinical records. In total, 365 HCC patients were included after the exclusion of individuals with inadequate survival data. Another RNA-sequencing dataset, ICGC-LIRI-JP, was employed for analysis. It contained 243 HCC samples that were retrieved from the ICGC database (https://dcc.icgc.org/). Detailed clinical data for all 365 TCGA-LIHC and 243 ICGC-LIRI-JP patients in our study were shown in the Supplementary Tables 1 and 2.

### Identification of mTORC1 signaling-related genes

To identify relevant gene sets related to mTORC1 signaling, we searched the Molecular Signatures Database (MSigDB; v7.5.1), which is a collection of annotated gene sets from published studies that comprise signaling cascades, biochemical pathways, expression profiling, and other biological concepts [20]. From the Molecular Signature Database (MSigDB; v7.5.1), 200 mTORC1 signaling-related genes were obtained from HALLMARK_MTORC1_SIGNALING. We applied GSVA function to evaluate gene activity of the mTORC1 signaling pathway in samples. Pearson correlation analysis was used to evaluate the correlation between gene expression and mTORC1 signaling pathway activity. Genes have been found to be correlated with mTORC1 pathway activity in both TCGA and ICGC databases with a |Cor| > 0.5 and *p*-value < 0.05. Those genes were utilized in further analysis.

### Identification of mTORC1 signaling-related subtypes

The consistency clustering “ConsensusClusterPlus” was employed to construct a consistency matrix, followed by clustering and typing of the samples [21]. The mTORC1 signaling-related signature subtypes of the sample were derived utilizing the chosen mTORC1 score-related genes. The Euclidean lows and Kaplan-Meier algorithms were employed for assessing distance, and bootstrap processes were carried out 500 times, each of which comprised 80% of the patients from the training set. The range for cluster sizes was set to be 2 to 10. Employing a consistency matrix as well as consistency cumulative distribution, optimal classification was discovered.

### Investigation of first-order partial correlation

To further screen for key genes significantly associated with the mTORC1 signaling pathway, a first-order partial correlation investigation was conducted using both the mTORC1 score and mTORC1 signaling pathway-related genes. mTORC1 score was set to x, and mTORC1 signaling-related genes levels were set to y. Under the gene condition, x and y constitute the following first-order partial correlation:


$$r_{xygene}\;=\;\frac{r_{xy}\;-\;r_{xgene}\;\times \;r_{ygene}}{\sqrt{\text{(}1-r_{xgene}^{2}\text{)}\;\times \;\text{(}1-r_{ygene}^{2}\text{)}\;}}$$

### Development and validation of the prognostic risk score

We conducted Cox regression analysis on the key genes identified through the above model screening. Using this analysis, we obtained a hazard ratio (HR) score for each gene. An equation was derived to calculate the risk score for individual patient using the formula as follows: Score = (beta I × Exp i), where beta i is the coefficient of mTORC1 signaling-related genes, and Exp i represent the expression of mTORC1 signaling-related genes. In the TCGA-LIHC cohort, each patient had a risk score on the basis of this signature, and every patient was divided into groups with high- or low-risk utilizing this score, using the threshold as the optimal segmentation point. Kaplan–Meier (K–M) survival analysis and log-rank test were utilized for examining differences (variations) in survival among the patients with high- and low-risk utilizing R statistical analysis software’s “survival” package to further investigate the signature’s prognostic significance. For examining the gene signature’s accuracy in anticipating patient prognosis, time-dependent receiver operating characteristic (ROC) curves were generated utilizing R’s “timeROC” package. Data from the ICGC cohort was used to validate the risk model, allowing for a further assessment of the robustness of the results.

### The immune score and mutation analysis

The “estimate” package was used to evaluate the tumor immune microenvironment score of cancer patients. Processing of bulk RNA-sequencing data allowed for the application of the deconvolution algorithm CIBERSORT [22] to compute the proportion of immune cells for each tumor subtype. The “maftools” package was used to obtain mutation data for TCGA-LIHC patients. The fisher’s exact test was used for significance testing of tumor mutation data.

### Evaluation of the TF activity

Utilizing the Garcia-Alonso approach, TFs’ activity was evaluated [23] and analysis of variance (ANOVA) was employed for the comparison of TF level activation across diverse groups.

### Functional enrichment analysis

Employing the clusterProfiler package, the Gene Ontology (GO) enrichment and Kyoto Encyclopedia of Genes and Genomes (KEGG) pathway analyses were executed. [24]. For false discovery rate (FDR) control, *Q*-value was derived to avoid a large FDR in repeated experiments. If a gene set had FDR < 0.05 and *p* < 0.05, it was considered substantially enriched.

### Treatment response prediction for immunotherapy

Multiple cancer treatments have been revolutionized by immune checkpoint inhibitor medications, and the TIDE algorithm (http://tide.dfci.harvard.edu/) [25] was employed to anticipate treatment outcomes of anti-programmed cell death 1 (PD-1) antibodies.

### Gene set enrichment analysis (GSEA)

The “ClusterProfiler” package was utilized for conducting GSEA by calculating the fold change values of all genes in both the high and low-risk groups. Additionally, we categorized the samples into two groups based on the median values of key genes and calculated the fold change values between these groups to perform GSEA analysis [24]. For GSEA, the gene sets form Hallmark and KEGG databases were utilized.

### Cell lines, and quantitative real-time polymerase chain reaction (PCR)

HepG2, Huh7, and Hep3B cell lines were purchased from the Guangzhou. Cellcook Biotech Co., Ltd (Guangdong, China). MHCC97L, MHCC97H, and HCCLM3 were gifted from Prof. Zhou Zhengjun, Fudan University, Zhongshan Hospital. Utilizing a TRIzol kit (Invitrogen, China), extraction of Total RNA from HCC and nontumor specimens was performed. Employing a reverse transcription kit, the synthesis of cDNA was performed. Real-time PCR tests were conducted in accordance with our prior literature employing the SYBR Premix Ex Taq kit (Takara, Dalian, China) following the kit’s instructions [26]. Utilizing the 2^−ΔΔCt^ technique, the gene’s relative quantification was evaluated. Following is a presentation of the primer sequences: PAG1 5′-TTCAGCCGTTCAGTTACTAGCC-3′ (forward) and 5′-TGGACTTCCTCGTAATGCTGC-3′ (reverse), LHFPL2 5′-CGGGTGAGTTGGGAATCAGA-3′ (forward) and 5′-CCTGCGGCCTCACCTATTTAT-3′ (reverse), FABP5 5′-TGAAAGAGCTAGGAGTAGGACTG-3′ (forward) and 5′-CTCTCGGTTTTGACCGTGATG-3′ (reverse), GAPDH 5′-GCCACATCGCTCAGACACCAT-3′, and 5′-CCCATACGACTGCAAAGACCC-3′. Rapamycin was used for inhibiting the mTORC1 signaling pathway, and HY-12028 and HY50846 were used for inhibiting the MAPK signaling pathway.

### Statistical analysis

R software (R Statistical Software, Austria) was employed to conduct statistical analyses. The statistical analysis was performed employing GraphPad Prism 8 for molecular biology verification. Unpaired *t*-tests were utilized for the comparison of the means between the two groups. All experimental results are presented as means ± SD and were repeated at least three times. Also, ^*^P < 0.05, ^**^P < 0.01, ^***^P < 0.001, and ^****^P < 0.0001 suggested a statistical significance level.

## RESULTS

### Identification of three critical mTORC1 signaling-related genes

**Figure 1:**
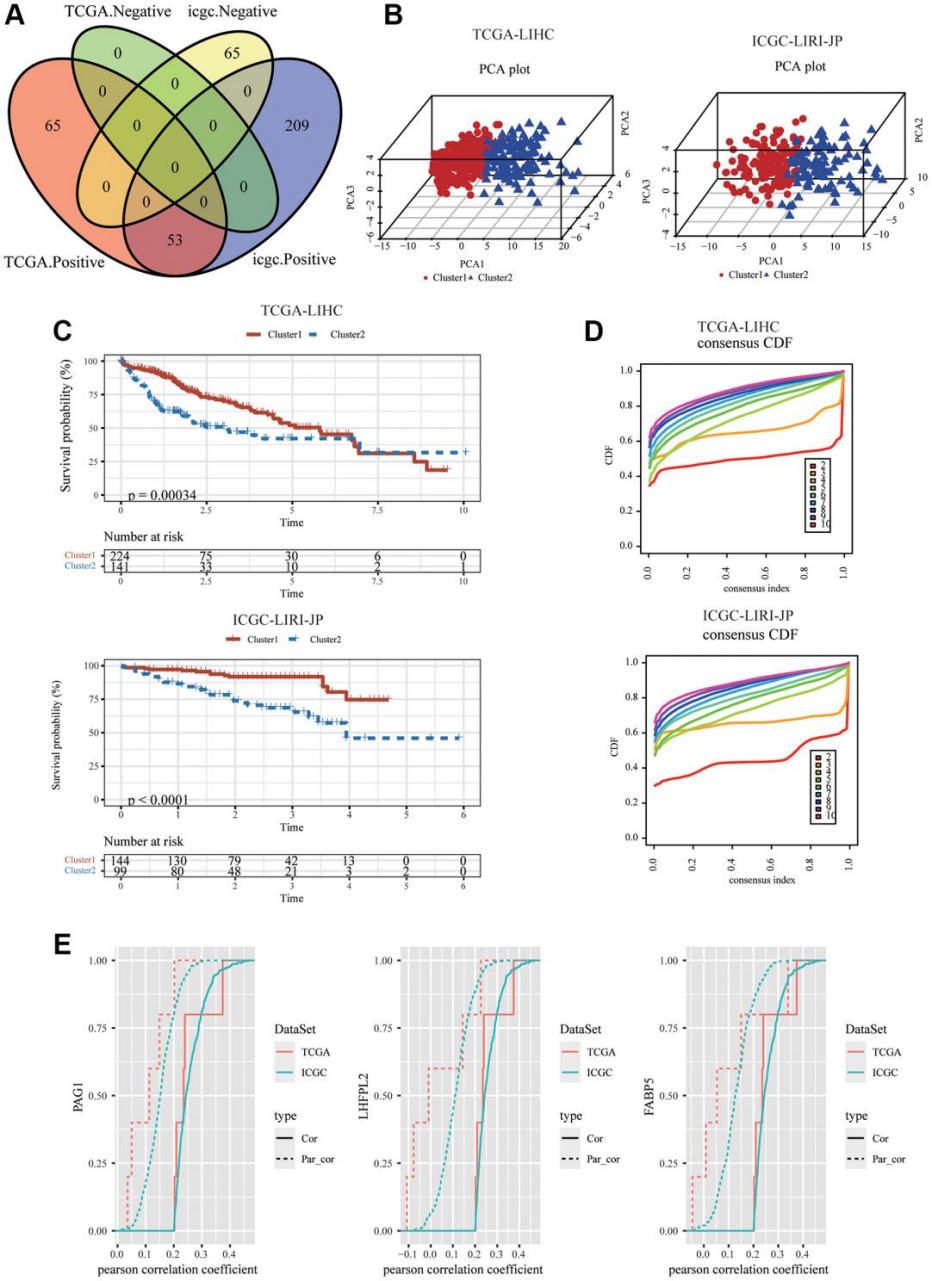
**Identification of the three HCC-specific mTORC1 signaling-related genes.** (**A**) Venn diagram showed that 53 mTORC1-related genes were positively correlated with mTORC1 scores in TCGC-LIHC and ICGC-LIRI-JP cohorts. (**B**) The 3D plots of PCA of TCGC-LIHC and ICGC-LIRI-JP dataset according to 53 genes’ expression levels in different risk groups. (**C**) The prognosis was significantly different between the two clusters in TCGC-LIHC and ICGC-LIRI-JP cohorts. (**D**) The relative alterations in the area under the CDF curves, and tracking plots exhibited the index from 2 to 9 in TCGC-LIHC and ICGC-LIRI-JP cohorts. (**E**) PAG1, LHFPL2, and FABP5 were identified as critical mTORC1-related genes, regardless of whether the first-order partial correlation is modified.

The workflow of the mTORC1 signaling-related genes signature analysis was shown in Supplementary Figure 1. To construct a mTORC1 signaling-related signature in HCC, 200 genes from the Hallmark mTORC1 pathway were extracted, and to derive a mTORC1 score for an individual patient, a single sample GESA was carried out. A total of 118 and 327 RNAs were substantially linked to mTORC1 scores (|Cor| > 0.5 and *P* < 0.05) from TCGA-LIHC and ICGC-LIRI-JP cohorts, correspondingly. Merely 53 genes from the two datasets overlapped and showed a positive correlation with mTORC1 scores (Figure 1A, Supplementary Figure 2). According to principal component analysis (PCA) analysis, the two clusters showed significant differences (Figure 1B). The results of the Kaplan–Meier plotter showed that these two clusters had different survival rates (Figure 1C). The optimal molecular clusters were then investigated employing the unsupervised machine learning algorithm, “ConsensusClusterPlus”. As depicted in Figure 1D, in the datasets ICGC-LIRI-JP and TCGA-LIHC; two clusters were shown to have the highest stability when measured utilizing the cumulative distribution function delta area curve. A first-order partial correlation investigation among mTORC1 scores and genes was carried out to identify the most significant genes in the mTORC1 pathway. Three key genes were more closely related to the mTORC1 pathway scores: Phosphoprotein Associated with Glycosphingolipid-Enriched Microdomains 1 (PAG1), LHFPL tetraspan subfamily member 2 (LHFPL2), and Fatty Acid Binding Protein 5 (FABP5) (Figure 1E).

### Construction and validation of a specific mTOR signature of HCC

**Figure 2:**
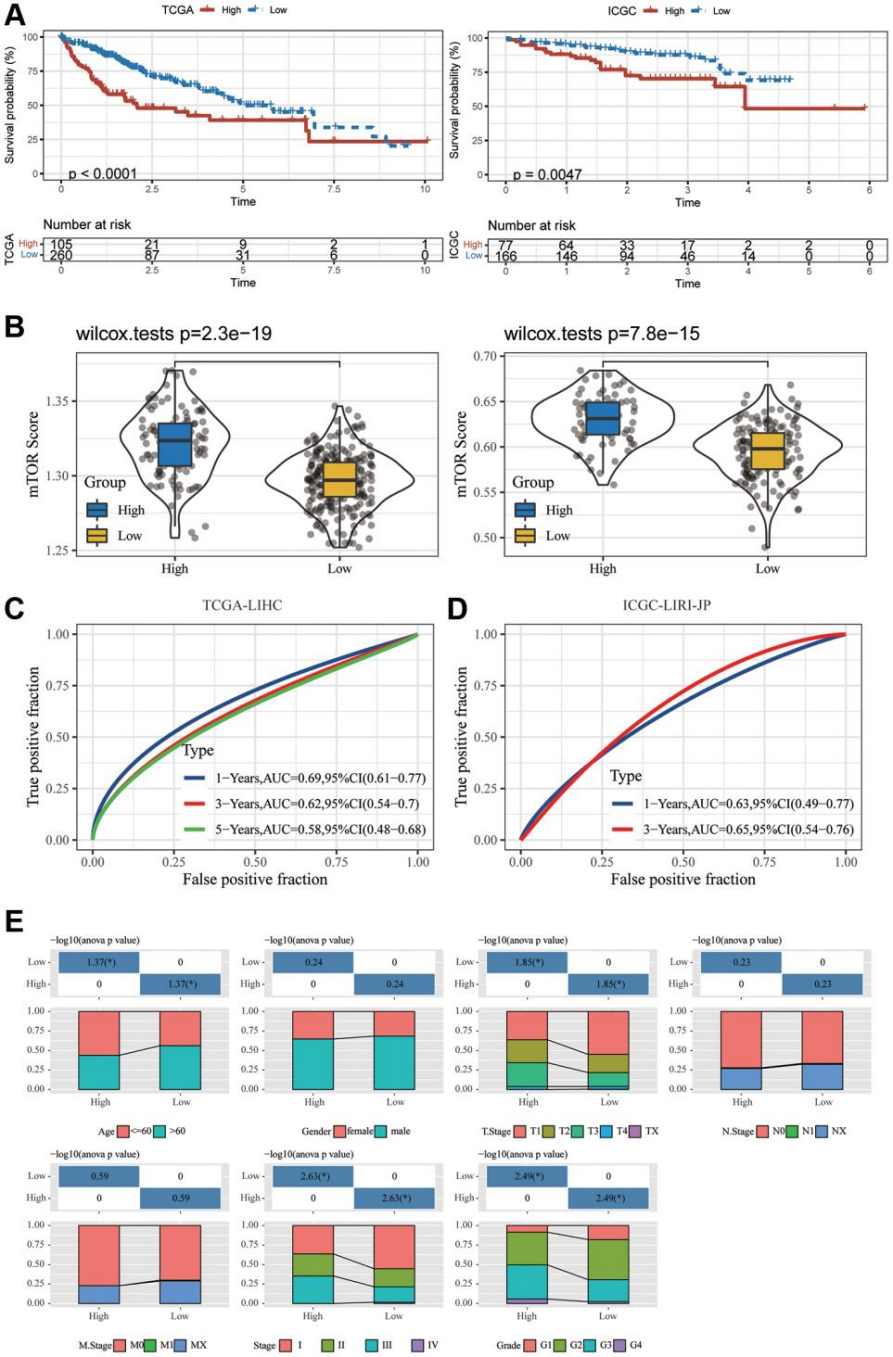
**Development and validation of an HCC-specific mTORC1 signaling-related signature.** (**A**) Kaplan–Meier plots indicate that the OS of patients in the group with high risk was substantially longer compared to those in the group with low risk in the TCGA-LIHC and ICGC-LIRI-JP cohorts. (**B**) The differences in the mTORC1-related score across groups with high and low risk in the TCGA-LIHC and ICGC-LIRI-JP cohorts. (**C**) ROC analysis of one-, three- and five-year OS signifying the excellent prognostic significance of mTORC1 score in the TCGA-LIHC cohorts. (**D**) ROC analysis of one-, and three-year OS signifying the excellent prognostic significance of mTORC1 score in the ICGC-LIRI-JP cohorts. (**E**) Variations across the groups with high and low risk in terms of various clinicopathological features.

Thus, we used the expression of PAG1, LHFPL2, and FABP5 to construct the model. For each tumor sample, a prognostic risk score was computed employing the equation below: prognostic risk score = PAG1 expression × 0.2399877 + LHFPL2 expression × 0.2969381 + FABP5 expression × 0.2441960 (TCGA). PAG1 × 0.06234738 + LHFPL2 expression × 0.30889841 + FABP5 expression × 0.13062274 (ICGC). The cut-off value for the prognostic risk score, which was computed to be 2.018198 (for TCGA) and 1.563421 (for ICGC), was utilized to classify patients into groups with high and low risk. In accordance with the Kaplan-Meier survival investigation, HCC patients in the group with a high expression of three genes had an inferior overall survival compared to those in the group with low expression (Figure 2A). ICGC-LIRI-JP dataset produced a similar outcome (Figure 2B). Subsequently, in ICGC-LIRI-JP and TCGA-LIHC datasets, the mTORC1 scores in the group with high risk were substantially increased when compared to that in the group with low risk (Figure 2C). A time-dependent ROC analysis was utilized for appraising the prognostic evaluation accuracy of mTOCR1-related signature. For the TCGA-LIHC dataset, Figure 2D exhibited that the area under the curve (AUC) at the one-, three-, and five-year OS reached 0.69, 0.62, and 0.58, respectively. In the ICGC-LIRI-JP dataset, the AUC for one-, and three-year OS reached 0.63 and 0.65, respectively, suggesting a powerful predictive value of the mTORC1-related signature. Age, grade, sex, and stage were the clinically pertinent characteristics. Moreover, we established a nomogram by combining mTORC1 score Gender, Age, Grade and TNM stage (Supplementary Figure 3) and found that mTORC1 score signature had the most significant influence on survival prediction. Furthermore, we observed that the prediction calibration curves of 1, 3, and 5 years were close to the standard curve, indicating that the nomogram had excellent performance. The result of ICGC-LIRI-JP dataset exhibited a similar outcome (Supplementary Figure 3). mTORC1 score variations stratified by several clinical features were compared. A high mTORC1 score was discovered in patients with younger age, worse pathological grade, more advanced stage of the tumor, as well as a higher TNM stage (Figure 2E). However, when the three genes are analyzed separately, their correlation with clinical indicators such as tumor size, *N* stage, M stage, and TNM stage is relatively poor (Supplementary Figure 4), which suggesting the superiority of the mTORC1 signaling-related signature.

### Pathway and mutations under distinct mTORC1 circumstances

**Figure 3:**
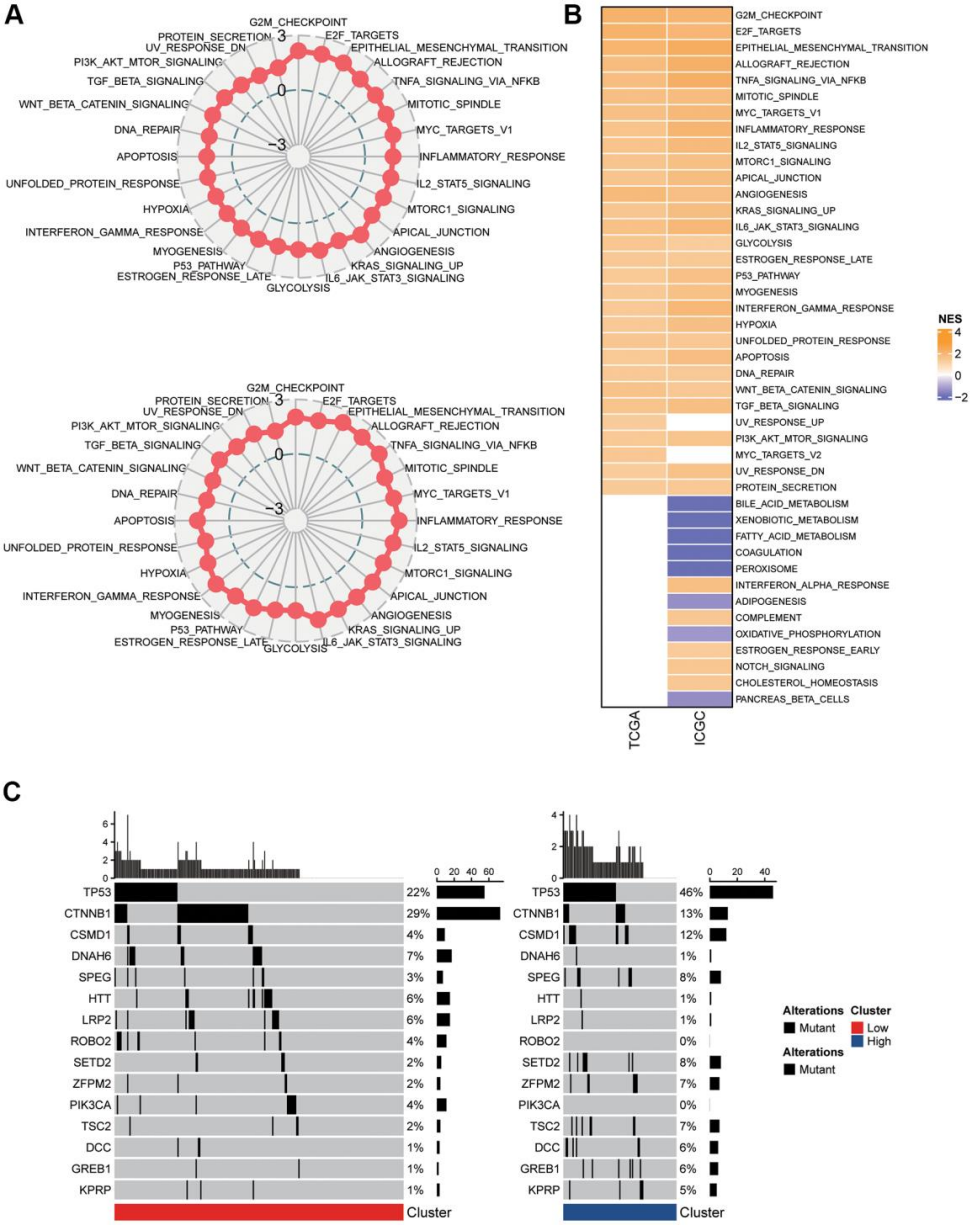
**Pathway analysis of the mTORC1 signaling-related signature.** (**A**) Radar plots demonstrating the NESs of the Hallmark pathways computed utilizing GSEA of C2 versus C1 in TCGA-LIHC and ICGC-LIRI-JP cohort. (**B**) A heatmap representing NESs of the Hallmark pathways computed through comparison of C2 with C1 in TCGA-LIHC and ICGC-LIRI-JP cohort. (**C**) Somatic mutation analysis of the two molecular subtypes.

In the two clusters, a differentially expressed gene (DEG) analysis and GSEA were carried out to study the activated cellular signaling pathways that underlie the mTORC1-associated genes. In the TCGA-LIHC dataset, 30 pathways were activated in C2 in comparison with the group with low risk; whereas in the ICGC-LIRI-JP dataset, 8 pathways were inhibited and 33 pathways were activated (Figure 3B). In total, 28 oncogenic pathways, including E2F targets, epithelial mesenchymal transition, G2M checkpoint, allograft rejection, inflammatory response, mitotic spindle, TNFA signaling via NFKB, apical junction, mTORC1 signaling, angiogenesis, glycolysis, estrogen response late, P53 pathway, myogenesis, interferon-gamma response, MYC targets, hypoxia, unfolded protein response, KRAS signaling up, IL-6 JAK STAT3 signaling, apoptosis, IL2 STAT5 signaling, DNA repair, WNT β-Catenin signaling, PI3K-Akt-mTOC signaling, UV response down, protein secretion, and TGF β signaling, overlapped across the two datasets (Figure 3A). Additionally, the differences were statistically substantial across C1 and C2 in terms of the nonsilent mutation rate (Figure 3C). Particularly, in TP53, CTNNB1, and CSMD1. The C1’s mutation burden was lower compared to C2. The mutation rates for TP53 (46% vs. 22%), and CSMD1 (12% vs. 4%), were greater in the C2 patients, while the mutation rate of CTNNB1 (13% vs. 29%) were lower in the C2 patients. Thus, the group with high risk demonstrated a more severe malignancy.

### Transcriptional factors profiling underlying risk scores

**Figure 4:**
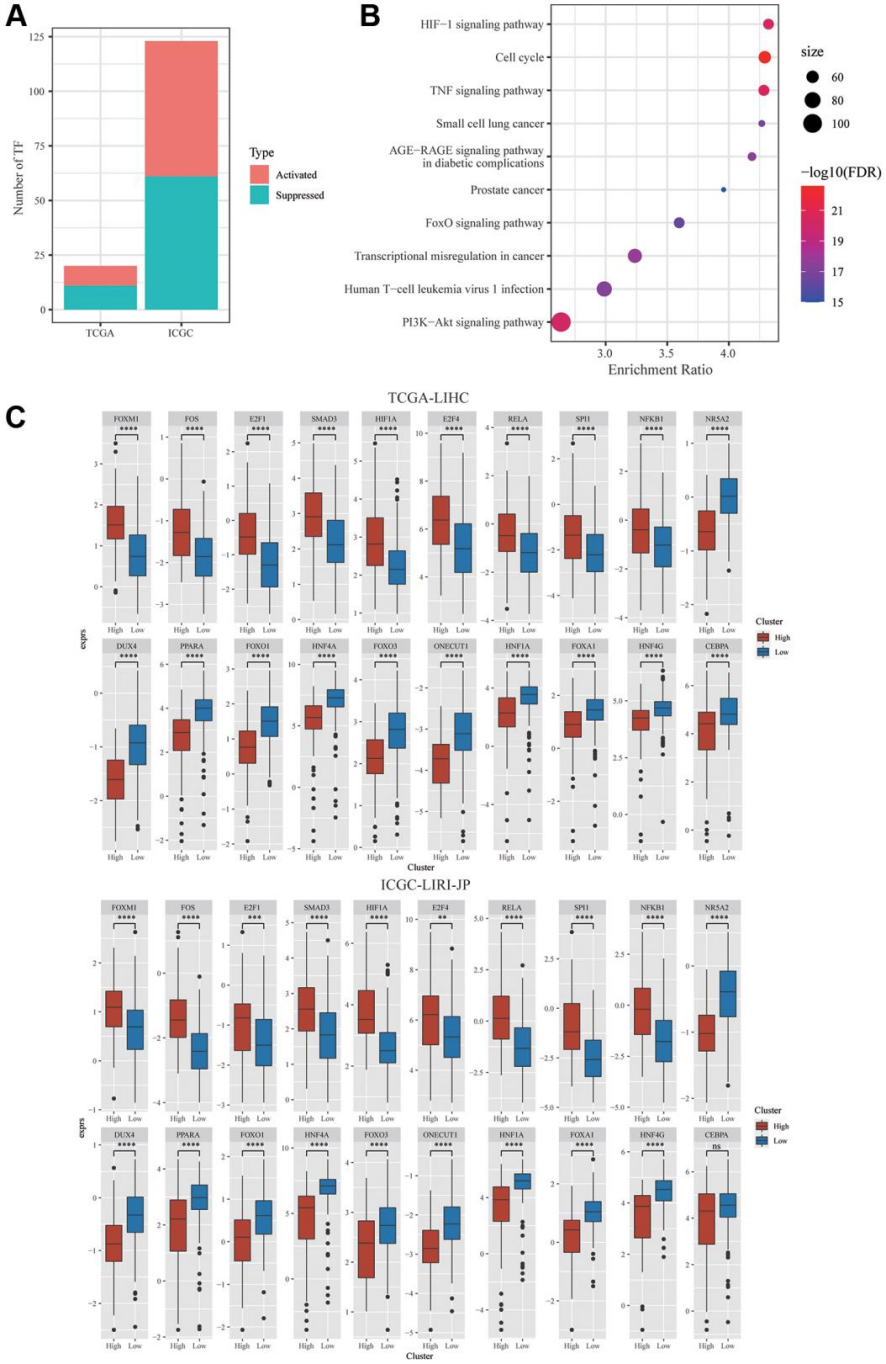
**Integrative Analysis of mTORC1-related genes.** (**A**) Distribution of the activated and suppressed TFs in C2 in comparison with C1 in TCGA-LIHC and ICGC-LIRI-JP cohort. (**B**) The findings of the functional enrichment analysis of TFs upregulated in the C1 subtype in TCGA-LIHC cohort. (**C**) The dysregulation in TF activity of the upregulated transcription factors in the TCGA-LIHC and ICGC-LIRI-JP cohort.

Furthermore, the relationship between dysregulated TFs and mTORC1 scores was investigated. Garcia-Alonso’s approach was employed to compute the TF activity score of individual samples from the ICGC-LIRI-JP and TCGA-LIHC datasets for the comparison of the activity of TF across two clusters. In TCGA-LIHC and ICGC-LIRI-JP cohorts, 9 and 62 substantially activated TFs, respectively, were found in the group with high risk. Furthermore, mTORC1 signaling-related genes and differentially expressed TFs had a substantially negative link among a set of TFs and a group of mTORC1-related genes, according to the analysis of this relationship (Figure 4A). In the TCGA-LIHC dataset, 11 TFs were substantially downregulated and 9 TFs were substantially upregulated in C2 in comparison with C1, whereas in the ICGC-LIRI-JP dataset, 61 TFs were substantially downregulated, whereas 62 TFs were substantially upregulated in C2 in comparison with C1 (Figure 4A). There was speculation that these 20 TFs were a factor in the poor prognosis of patients in the C2 cluster. Analysis of the 20 TFs’ cellular signaling pathway enrichment was carried out to validate this hypothesis. The findings exhibited that a few crucial cancer-related pathways, including TNF signaling pathway, cell cycle, HIF-1Alpha signaling pathway, IL-17 signaling pathway, and PI3K-Akt signaling pathway were considerably enhanced in the TFs’ downstream targets (Figure 4B). Furthermore, in the TCGA dataset, nine TFs’ expression levels were substantially elevated, and the expression levels of eleven TFs were substantially lowered in C2 in comparison with C1 (Figure 4C). Thus, the combination of these mTORC1 signaling-related genes and TFs may facilitate the development of HCC.

### Immune cell features and purposes in diverse prognostic risk scores

**Figure 5:**
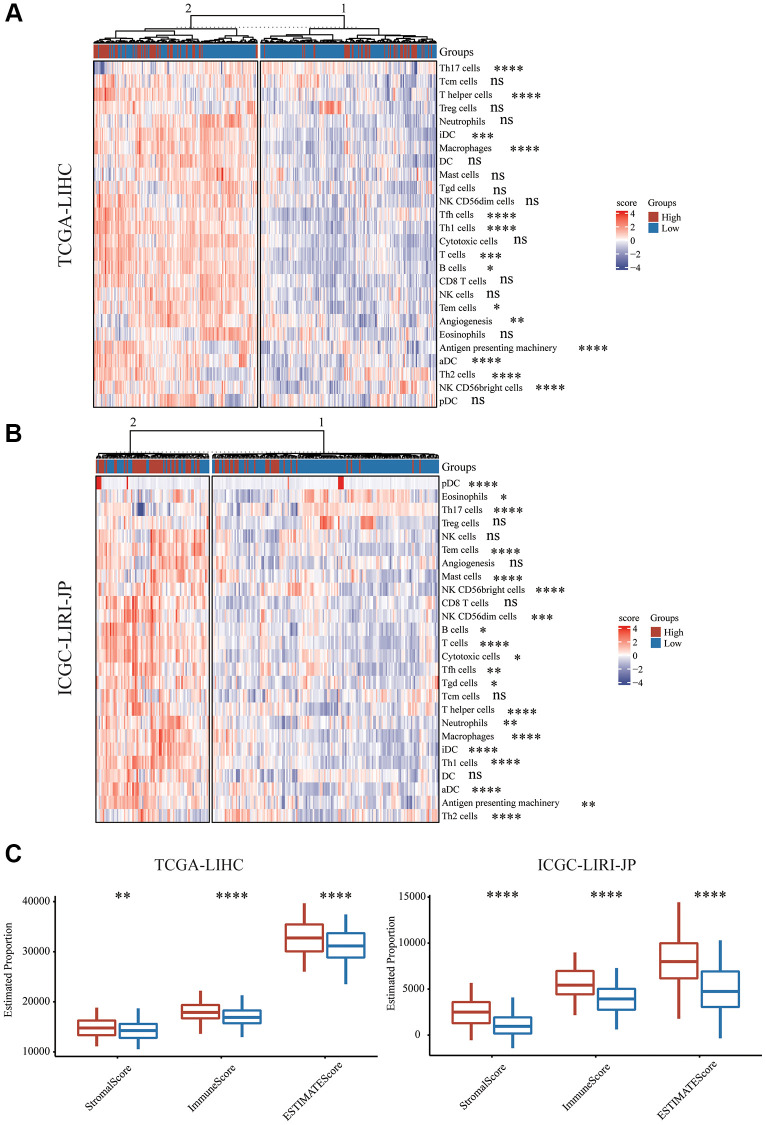
**Immune characteristics of mTORC1 signaling-related subgroups.** (**A**) Variations in immune infiltration scores were evaluated in TCGA-LIHC cohort. (**B**) The variation in multiple immune infiltration scores was assessed in ICGC-LIRI-JP cohort. (**C**) Immune score, stromal score and ESTIMATE score differences across the group with high and low risk.

Utilizing immune cell marker gene expression, the level of immune cell infiltration in the HCC cohorts was assessed in order to further investigate the immune microenvironment variations across the two subgroups. Prior studies served as the source for the immune cell marker genes [27]. CD8 T cells are the most important cell type. The CD8 T cells in the two groups did not substantially differ from one another. It was discovered that Th17 cells had a greater abundance in the group with low risk, though T cells, T helper cells, Tfh cells, Th1 cells, and Th2 cells had a greater abundance in the group with high risk (Figure 5A, 5B). Additionally, infiltrating NK cell subtype in the group with high risk was primarily associated with NK CD56 bright cells, while NK cells and NK CD56 dim cells exhibited no significant difference (Figure 5A, 5B). The immune functions between the different risk scores were additionally investigated. Further immunosuppressive properties were discovered in the group with high risk, including aDC, iDC, B cell, and Macrophage. Subsequently, different risk scores’ immune cell composition and correlation were examined. We used an immune score to detect the microenvironment. We utilized the ESTIMATE algorithm for the assessment of the immune score and stromal score of HCC and discovered that the stromal score, immune score, and ESTIMATED score in the group with high risk were substantially elevated in comparison with those in the group with low risk (Figure 5C).

### Prospective predictive value of mTORC1 signature for immunotherapy response

**Figure 6:**
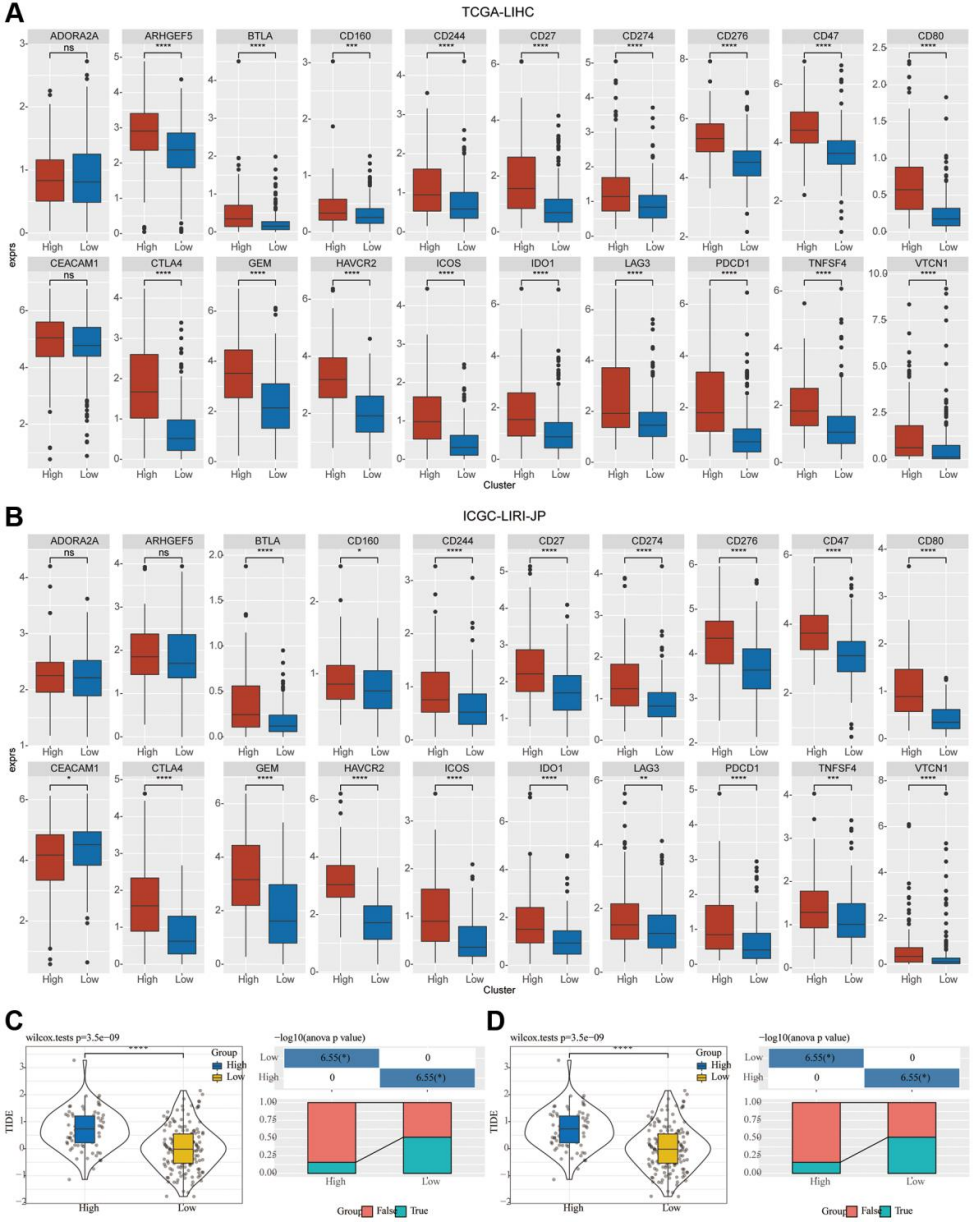
**Investigation of the variation in immunotherapy across mTORC1-related subgroups.** (**A**, **B**) The boxplots illustrate the immune checkpoints that were upregulated in C1 in comparison with C2 in TCGA-LIHC and ICGC-LIRI-JP cohorts. (**C**) Variations in the TIDE score and immune response status of the diverse molecular subtypes in the TCGA-LIHC cohort. (**D**) Variations in the TIDE score and immune response status of the diverse molecular subtypes in the ICGC-LIRI-JP cohort.

Immune checkpoint genes in the HisgAtalas dataset were examined in light of the immunotherapy’s significance in the treatment of HCC. The findings exhibited that multiple immune checkpoint genes were substantially enhanced in C2 compared to C1. Multiple crucial immune checkpoint genes had elevated levels of expression in the high-risk subgroup of C2, including BTLA, CD27, CD47, CD80, CD160, CD244, CD274, CD276, CTLA4, GEM, HAVCR2, ICOS1, IDO1, LAG3, PDCD1, TNFSF4, and VTCN1 in comparison to those of C1 (Figure 6A, 6B). Furthermore, in the two clusters, the potential response to immunotherapy was evaluated by employing the tumor Immune dysfunction and exclusion (TIDE) software. Immune escape is more likely to occur with a higher TIDE prediction score, which suggests that immunotherapy is less likely to be beneficial for such patients (Figure 6C, 6D). In the TCGA cohort, TIDE scores in C2 were greater compared to those in C1, indicating that patients in C2 may be more susceptible to immune escape and experience fewer immunotherapy benefits (Figure 6C). In the ICGC-LIRI-JP group, a comparable difference was seen in the immunotherapy response’s predictive value (Figure 6D).

### Molecular pathways and experimental verification of the three genes

**Figure 7:**
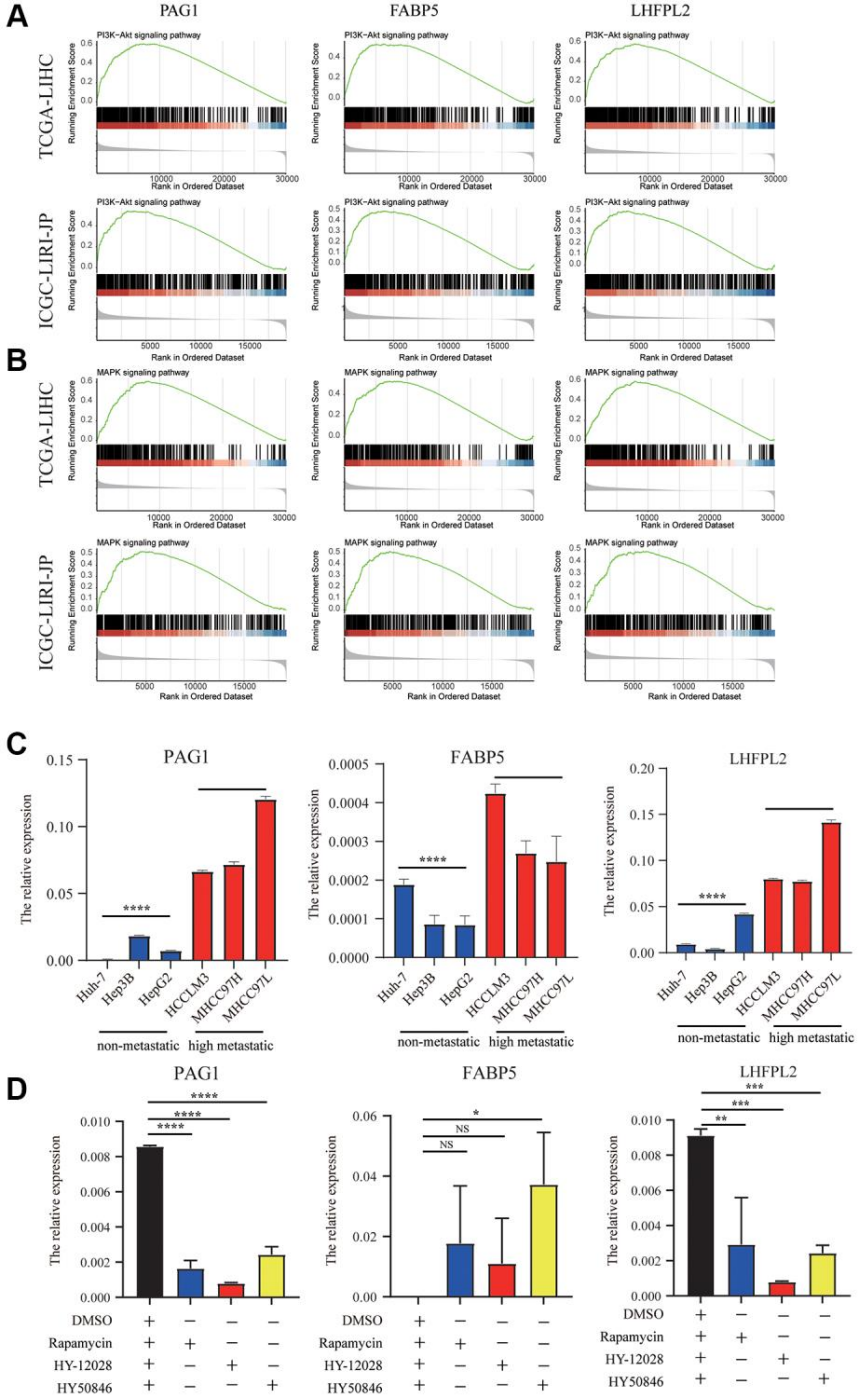
**Molecular pathways and experimental verification of the three genes.** (**A**) PI3K-AKT signaling was enhanced in the high-expression group in TCGA-LIHC and ICGC-LIRI-JP datasets. (**B**) MAPK pathways was significantly correlated with our model. (**C**) The expression of PAG1, LHFPL2, and FABP5 in high HCC metastatic cell lines were higher than those in HCC non-metastatic cell lines. (**D**) The expression of PAG1 and LHFPL2 were significantly downregulated in MHCC97H cell lines, whereas the expression of FABP5 was enhanced in MHCC97H cell lines after treating with mTORC1 blocker or MEK/ERK inhibitors.

According to GSEA analysis, PI3K-AKT signaling, were primarily enhanced in the high-expression group in TCGA-LIHC and ICGC-LIRI-JP datasets (Figure 7A). Surprisingly, we found that MAPK pathways was significantly correlated with our model (Figure 7B). Additionally, we utilized cell line experiments to further confirm the findings in the database. It was found that the details of the expression of PAG1, LHFPL2, and FABP5 in high HCC metastatic cell lines were substantially greater in comparison with those in HCC non-metastatic cell lines (Figure 7C). Subsequently, following incubation with mTORC1 blocker or MEK/ERK inhibitors for 48 h, the expression of PAG1 and LHFPL2 were significantly downregulated in MHCC97H cell lines, whereas the expression of FABP5 was enhanced in MHCC97H cell lines (Figure 7D).

## DISCUSSION

The onset and advancement of HCC are strongly influenced by genetic factors. Signature developed based on specific characteristics including glycolysis and immunity has been found and may help anticipate the prognosis in cancer patients. A novel gene signature was developed in the current work based on particular genes linked to the mTORC1 signaling pathway that can anticipate HCC patients’ prognoses. As a result, finding prognostic genes linked to the mTORC1 signaling pathway is crucial for directing future investigation into treatment strategies for HCC patients.

In our investigation, the datasets TCGA-LIHC and ICGC-LIRI-JP contained 53 genes that were found to be associated with the mTORC1 signaling pathway. Subsequently, based on the first-order partial correlation investigation, we discovered three crucial genes for developing the signature. HCC patients who had high levels of PAG1, LHFPL2, and FABP5 expression, had a worse prognosis. PAG1 is one of the membrane proteins that is more frequently tyrosine-phosphorylated in the control of T-cell activation [28, 29]. PAG is dephosphorylated after activation via the T cell receptor (TCR), which enables the release of CSK from the membrane and SRC inhibition [30, 31]. PAG1 is closely linked to human malignancies. According to a recent study, PAG expression quantitative trait loci (eQTL) are substantially linked to melanoma patient survival and treatment responsiveness [32]. Colon cancer and melanoma tumors are more susceptible to PD-1 inhibition when PAG1 is deleted [33]. The family of genes known as the lipoma HMGIC fusion partner (LHFP) includes the LHFPL tetraspan subfamily member 2 (LHFPL2). In cases of acute myeloid leukemia and triple-negative breast cancer, it is linked to chronic proliferation and macrophages, respectively [34]. Previous literature has shown that LHFPL2 was involved in the tumor microenvironment-related signature [35]. FABP5 (fatty acid binding protein 5) transports lipids inside cells where they are stored. Another research shows that HDL function may be directly regulated by circulating FABP5, regardless of HDL cholesterol levels, and that cholesterol efflux in macrophages may be decreased [36]. In HCC, FABP5 was found to enhance angiogenesis [37]. According to these findings, FABP5 may be involved in HCC and cholesterol homeostasis. FAPB5 also have been reported in the cholesterol metabolism-related signature in HCC [38]. Activation of FABP5 increases immune tolerance formation by the accumulation of lipid droplets and upregulation of PD-L1. The overexpression of FABP5 in monocytes was associated with HCC patients’ worse OS time [39]. Furthermore, we conducted RT-PCR analyses and conclude that mRNA expression levels of PAG1, LHFPL2, and FABP5 were elevated in the HCC metastatic cell lines, as compared to the low metastatic cell lines. Moreover, the PAG1 and LHFPL2 expression levels were remarkably linked to the MAPK and PI3K/AKT signaling. The link between PAG1, LHFPL2, and FABP5 and MAPK and PI3K/AKT signaling was limited. Pervious reported that the expression of PAG1 was suppressed by inhibiting MEK and PI3K [40]. To enhance mTORC1 activation, Ras/MAPK activation triggers ERK-dependent Raptor phosphorylation [41]. Nevertheless, it is yet unknown how FABP5 influences HCC through cholesterol metabolism, which necessitates more research.

The majority of cancers are known to be heterogeneous, and the same applies to HCC. One of the best strategies to increase patient survival would surely be to make a substantial assessment of tumor heterogeneity. To some extent, gene expression levels-based prognostic models can compensate for TNM staging’s limitations in anticipating patients’ prognoses. The signature developed in this research performed well in anticipating HCC patients’ prognoses. Moreover, we found that the enrichment of TFs underlying mTOR signaling-related signature is the HIF-1Alpha signaling pathway, TNF signaling pathway, and cell cycle. In the two datasets, nine TFs’ expression levels were substantially elevated, and eleven TFs’ expression levels were substantially lowered in C2 when compared to C1. These TFs, along with mTORC1, contribute to HCC progression.

Since risk scores related to mTORC1 signaling were linked to an inferior prognosis in HCC individuals, the link between risk scores and PD-1 treatment resistance was investigated and directed. Immunotherapy can be improved after comprehending immune cell composition, molecular mechanisms, and immunological roles of various risk scores in TME. Utilizing CIBERSORT, the correlation among infiltration immune cells was evaluated, and the results were related to various mTORC1-related risk scores. To maintain immune cell function, mTORC1 activity is crucial, and the immune response can be considerably changed or completely eradicated when mTORC1 is inhibited. For normalizing tumor blood vessels, prior research discovered that mTORC1 was a good target, which might strengthen antitumor immunity. The immune microenvironment around the tumor tissues is reflected by tumor immune cell infiltration, which may affect patients’ response to immunotherapy. PD-1 blockade is also being acknowledged as a model for the successful treatment of cancer [42]. In the current research, the infiltration status of immunocytes across the two risk groups was compared, and we also evaluated the therapeutic variations in the plethora of immune infiltrating cells comprising T cells, Tfh cells, T helper cells, Th1 cells, and Th2 cells across the high- and low- risk groups. In light of the alterations in the tumor’s immune microenvironment, this discovery elucidates the variation in the prognosis across different HCC patients. Finally, when the capacity to anticipate immunotherapy reactions was compared across the two groups, it was shown that patients in the low-risk group benefited further from anti-PD-1 therapy, which may help to direct clinical treatment.

The development of a novel and potentially useful tool that can help determine people who would gain the most benefit from immunotherapy as well as predict HCC patients’ prognosis is the most significant contribution of this research. The current research, however, has certain confines. First, the bioinformatics analysis for the mTORC1 signaling model needs to be validated in multiple centers. Second, there is relatively little clinical data available from public sources, and it is unidentified if the patient has further metabolic disorders. Third, the potential mechanisms of the three genes in the tumor microenvironment during HCC progression were not entirely understood. Future research will focus on the clinical utility of this predictive risk model, and a number of *in vivo* and *in vitro* laboratory tests will be conducted.

## CONCLUSIONS

Based on mTORC1 signaling-related genes, we have constructed a prognosis risk signature that can predict OS in HCC patients. This model has the potential to be a useful tool for predicting immune cell levels and patients’ sensitivity to immunotherapy.

## Supplementary Materials

Supplementary Figures

Supplementary Tables
