# Supplementary material for: A novel hepatocellular carcinoma-specific mTORC1-related signature for anticipating prognosis and immunotherapy
Source: Aging (Albany NY). 2023 Aug 16;15(16):7933–55. doi: 10.18632/aging.204862 (PMC10497017; doi:10.18632/aging.204862)
Supplement: Supplementary Figures [file aging-15-204862-s001.pdf]

SUPPLEMENTARY FIGURES

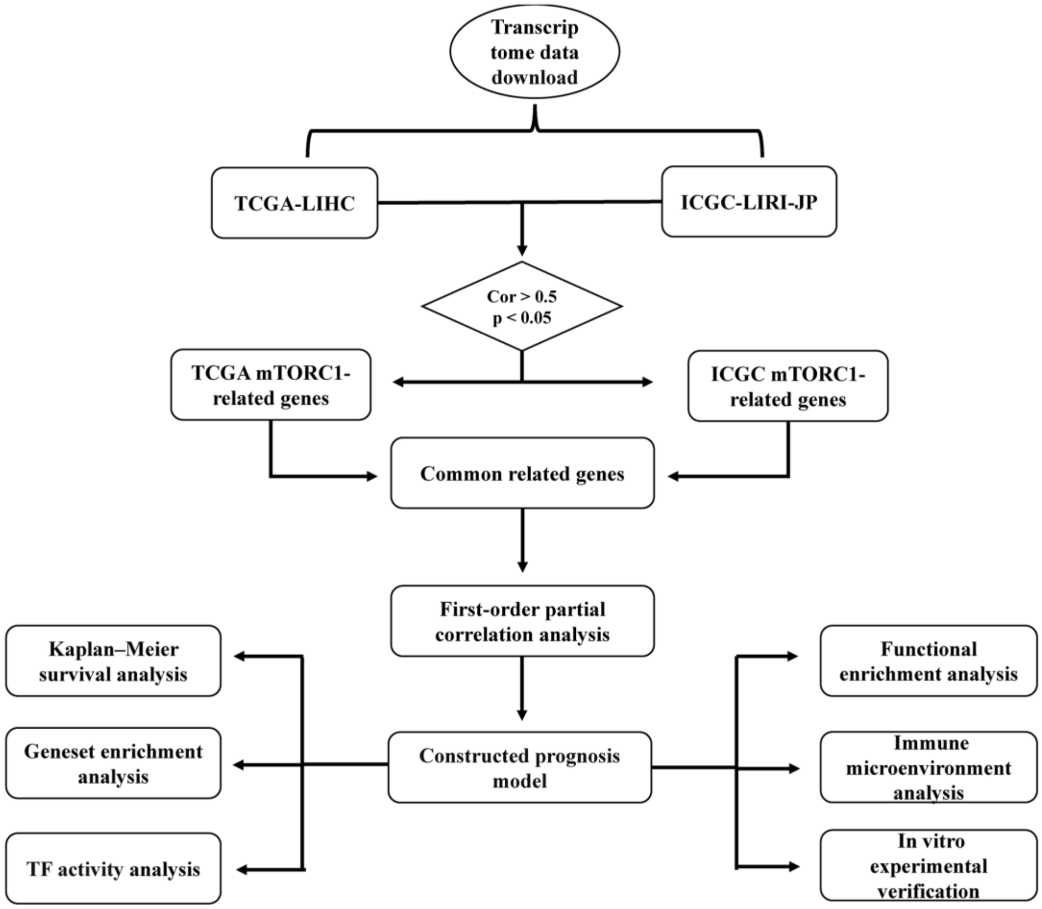

Supplementary Figure 1. Flow diagram of the study.

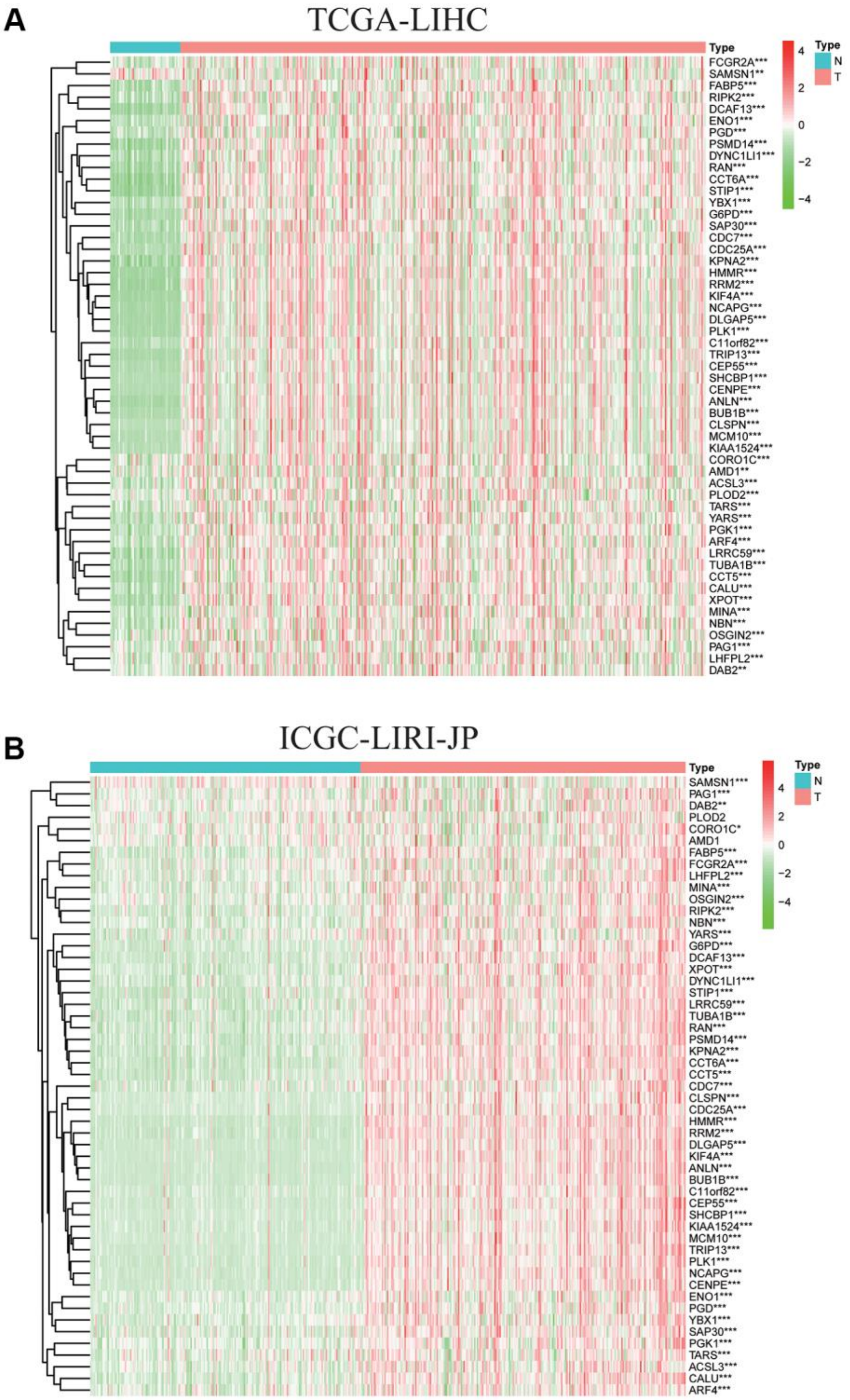

Supplementary Figure 2. Heatmap of the mRNA expression levels of 53 genes in the two subtypes in (**A**) TCGA-LIHC and (**B**) ICGC-LIRI-JP datasets.

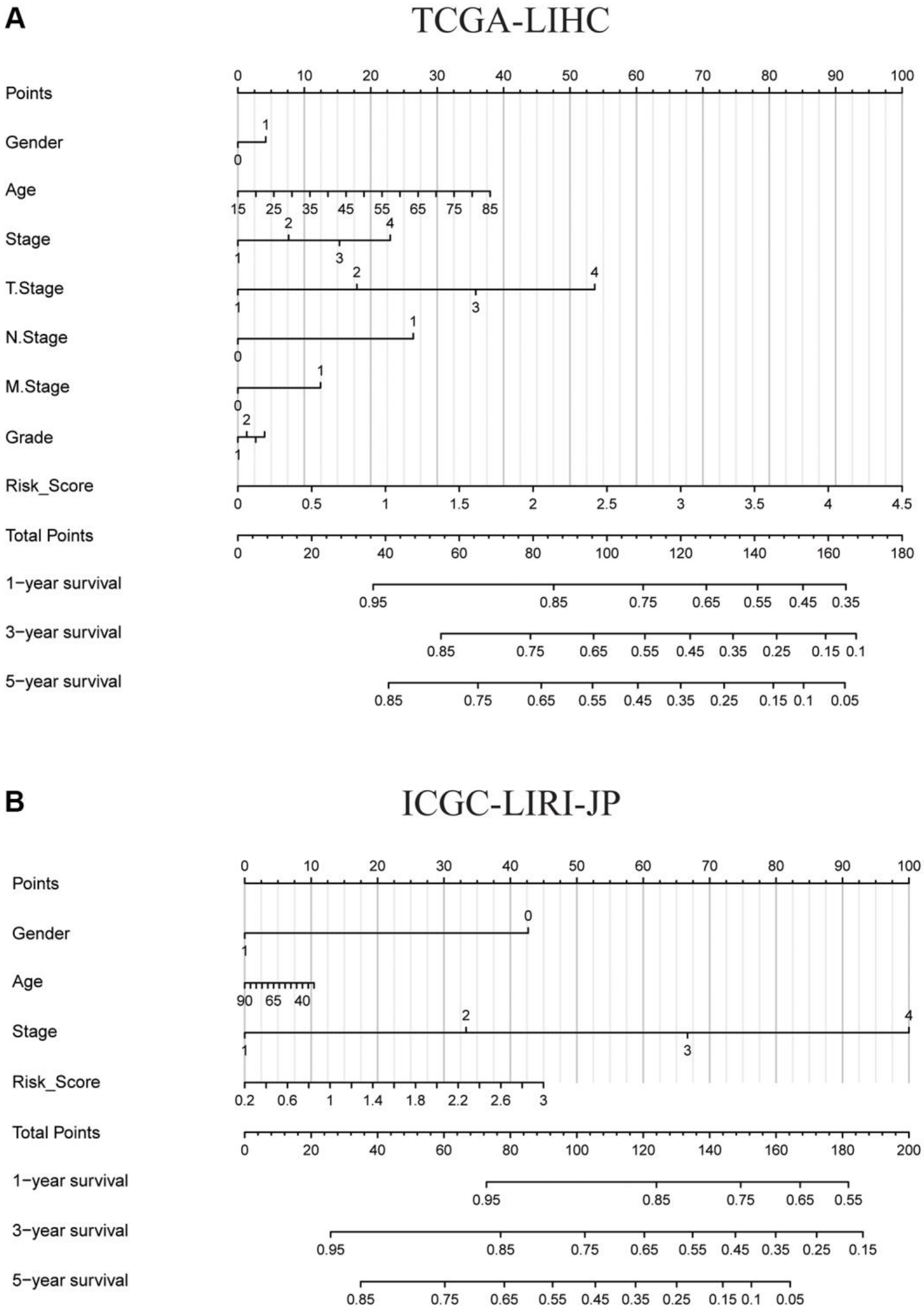

Supplementary Figure 3. Construction of nomogram to assess the predictive efficiency of mTORC1-related signature in (**A**) TCGA-LIHC and (**B**) ICGC-LIRI-JP datasets.

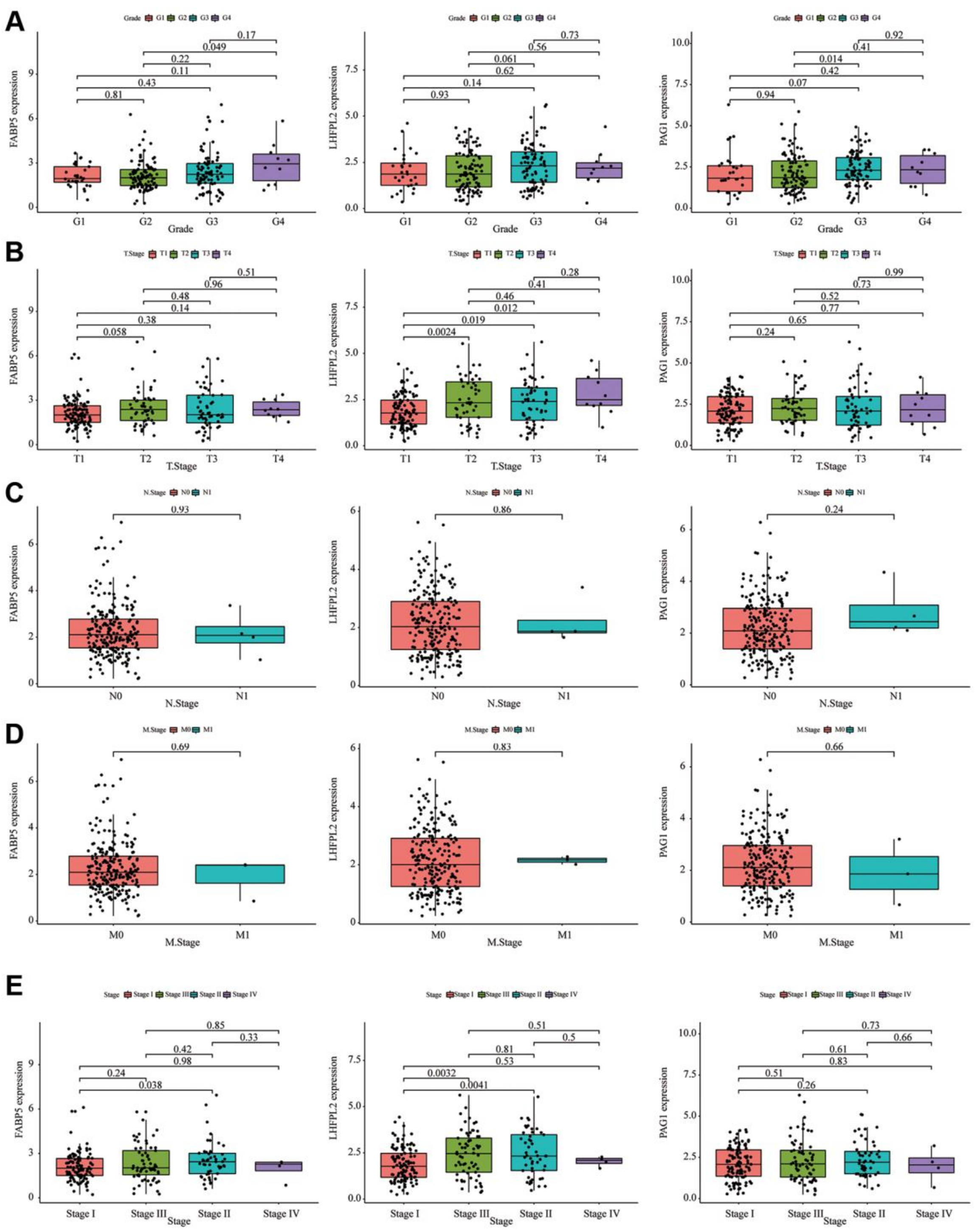

Supplementary Figure 4. (**A**) The expression levels of three genes in different grades of cancers from TCGA database. (**B**) The expression levels of three genes in different T stages of cancers from TCGA database. (**C**) The expression levels of three genes in different *N* stages of cancers from TCGA database. (**D**) The expression levels of three genes in different M stages of cancers from TCGA database. (**E**) The expression levels of three genes in different tumor stages of cancers from TCGA database.
