# Supplementary material for: A novel hepatocellular carcinoma-specific mTORC1-related signature for anticipating prognosis and immunotherapy
Source: Aging (Albany NY). 2023 Aug 16;15(16):7933–55. doi: 10.18632/aging.204862 (PMC10497017; doi:10.18632/aging.204862)
Supplement: Supplementary Tables [file aging-15-204862-s002.pdf]

## SUPPLEMENTARY TABLES

**Supplementary Table 1. Detailed clinical data for all 365 TCGA-LIHC patients.**

| | High (*N* = 105) | Low (*N* = 260) | *P* value |
|---|---|---|---|
| OS: | | | 0.002 |
| Alive | 54 (51.4%) | 181 (69.6%) | |
| Dead | 51 (48.6%) | 79 (30.4%) | |
| Gender: | | | 0.576 |
| Female | 37 (35.2%) | 82 (31.5%) | |
| Male | 68 (64.8%) | 178 (68.5%) | |
| Age: | | | 0.043 |
| ≤60 | 59 (56.2%) | 114 (43.8%) | |
| >60 | 46 (43.8%) | 146 (56.2%) | |
| Stage: | | | 0.002 |
| Stage I | 36 (36.4%) | 134 (55.4%) | |
| Stage II | 28 (28.3%) | 56 (23.1%) | |
| Stage III | 35 (35.4%) | 48 (19.8%) | |
| Stage IV | 0 (0.00%) | 4 (1.65%) | |
| Grade: | | | 0.003 |
| G1 | 9 (8.57%) | 46 (18.0%) | |
| G2 | 44 (41.9%) | 131 (51.4%) | |
| G3 | 46 (43.8%) | 72 (28.2%) | |
| G4 | 6 (5.71%) | 6 (2.35%) | |
| T. Stage: | | | 0.008 |
| T1 | 38 (36.2%) | 142 (55.0%) | |
| T2 | 31 (29.5%) | 60 (23.3%) | |
| T3 | 32 (30.5%) | 46 (17.8%) | |
| T4 | 4 (3.81%) | 9 (3.49%) | |
| TX | 0 (0.00%) | 1 (0.39%) | |
| *N*. Stage: | | | 0.688 |
| N0 | 75 (72.1%) | 173 (66.5%) | |
| N1 | 1 (0.96%) | 3 (1.15%) | |
| NX | 28 (26.9%) | 84 (32.3%) | |
| M. Stage: | | | 0.354 |
| M0 | 81 (77.1%) | 182 (70.0%) | |
| M1 | 0 (0.00%) | 3 (1.15%) | |
| MX | 24 (22.9%) | 75 (28.8%) | |
| mTOR | 1.32 [1.31;1.34] | 1.30 [1.29;1.31] | <0.001 |

**Supplementary Table 2. Detailed clinical data for 243 ICGC-LIRI-JP patients.**

| | High (*N* = 77) | Low (*N* = 166) | *P* value |
|---|---|---|---|
| OS: | | | 0.019 |
| Alive | 56 (72.7%) | 143 (86.1%) | |
| Dead | 21 (27.3%) | 23 (13.9%) | |
| Gender: | | | 0.957 |
| Female | 20 (26.0%) | 41 (24.7%) | |
| Male | 57 (74.0%) | 125 (75.3%) | |
| Age: | | | 0.823 |
| ≤60 | 17 (22.1%) | 33 (19.9%) | |
| >60 | 60 (77.9%) | 133 (80.1%) | |
| Stage: | | | 0.375 |
| Stage I | 10 (13.0%) | 26 (15.7%) | |
| Stage II | 30 (39.0%) | 80 (48.2%) | |
| Stage III | 29 (37.7%) | 47 (28.3%) | |
| Stage IV | 8 (10.4%) | 13 (7.83%) | |
| mTOR | 0.63 (0.03) | 0.60 (0.03) | <0.001 |
